# SIRT3 deficiency aggravates renal injury and fibrosis in chronic kidney disease and is associated with intestinal barrier dysfunction and gut microbiota dysbiosis

**DOI:** 10.3389/fmicb.2026.1851246

**Published:** 2026-07-13

**Authors:** Zhili Xiong, Rong Qian, Xiaoyu Zhang, Rui Wang, Mengdi Cui, Huiying Xiong, Xue Tian, Bing Guan

**Affiliations:** 1 Hubei Provincial Hospital of Traditional Chinese Medicine, Wuhan, China; 2 Affiliated Hospital of Hubei University of Chinese Medicine, Wuhan, China; 3 Hubei Research Institute of Traditional Chinese Medicine, Wuhan, China; 4 Hubei Key Laboratory of Theory and Application Research of Liver and Kidney in Traditional Chinese Medicine, Wuhan, China

**Keywords:** chronic kidney disease, gut microbiota dysbiosis, intestinal barrier dysfunction, renal fibrosis, SIRT3

## Abstract

**Background:**

Chronic kidney disease (CKD) is a progressive disorder characterized by persistent renal dysfunction, inflammation, and fibrosis. Sirtuin 3 (SIRT3), a mitochondrial nicotinamide adenine dinucleotide-dependent deacetylase, has been implicated in the regulation of mitochondrial homeostasis, oxidative stress, and renal injury. However, whether SIRT3 participates in CKD progression through gut–kidney axis-related alterations remains unclear. This study investigated the role of SIRT3 in renal injury, fibrosis, intestinal barrier dysfunction, and gut microbiota dysbiosis in CKD.

**Methods:**

An adenine-induced CKD model was established in global SIRT3 knockout mice. Renal function, inflammatory markers, histopathological injury, fibrosis-related proteins, colonic injury, intestinal barrier-related proteins, and gut microbial composition were evaluated. In parallel, human proximal tubular epithelial HK-2 cells were treated with transforming growth factor-β1 (TGF-β1), with or without the SIRT3 inhibitor 3-TYP, to assess profibrotic responses and apoptosis *in vitro*. Gut microbiota alterations were analyzed by 16S rRNA gene sequencing.

**Results:**

SIRT3 deficiency alone did not produce overt abnormalities under basal conditions, but significantly aggravated adenine-induced renal dysfunction, inflammatory responses, tubulointerstitial fibrosis, and structural kidney injury. In CKD mice, SIRT3 deficiency also worsened colonic injury, reduced the expression of zonula occludens-1 and occludin, and was associated with more pronounced gut microbiota dysbiosis. *In vitro*, inhibition of SIRT3 further enhanced TGF-β1-induced profibrotic protein expression and apoptosis in HK-2 cells. Correlation analysis further suggested significant associations between specific microbial taxa and indices of renal fibrosis and renal dysfunction.

**Discussion:**

These findings indicate that SIRT3 exerts a protective role in CKD progression. Loss of SIRT3 aggravates CKD progression not only by worsening local renal injury, but also in association with gut–kidney axis-related abnormalities. Collectively, our results suggest that SIRT3 may represent a potential therapeutic target for CKD and provide a basis for further mechanistic investigation of its role in renal fibrosis and gut–kidney axis dysfunction

## 1 Introduction

Chronic kidney disease (CKD) is a progressive disorder characterized by structural or functional abnormalities of the kidney that persist for more than 3 months and adversely affect health, typically presenting as a reduction in estimated glomerular filtration rate (eGFR) and increased urinary albumin excretion (KDIGO, 2024). CKD has become a major global public health challenge and a leading cause of mortality and disability. According to the 2023 Global Burden of Disease study, approximately 788 million adults aged 20 years or older were living with CKD worldwide in 2023, corresponding to an age-standardized prevalence of 14.2%. CKD has also become the ninth leading cause of death worldwide, accounting for approximately 1.48 million deaths. Notably, impaired kidney function is an important systemic risk factor and is associated with 11.5% of cardiovascular deaths globally (Mark et al., 2025). Although therapeutic strategies such as renin–angiotensin–aldosterone system (RAAS) blockade are widely used in clinical practice, current interventions remain insufficient to fully prevent ongoing kidney injury and progressive fibrosis in the context of a multifactorial CKD burden driven by hyperglycemia, hypertension, and elevated body mass index (Francis et al., 2024). Identifying novel therapeutic targets capable of modulating the complex pathogenic network of CKD therefore remains a major priority in both basic and clinical research.

Sirtuins are nicotinamide adenine dinucleotide (NAD+)-dependent deacetylases and deacylases that play essential roles in the regulation of energy metabolism, stress responses, and cell fate (Peng et al., 2024). Among them, SIRT3 is predominantly localized to mitochondria and is widely recognized as a critical regulator of mitochondrial homeostasis, redox balance, and energy metabolism (Azzouz and Yan, 2025). In the kidney, a highly energy-demanding organ, SIRT3 protects against tubular epithelial injury and renal fibrogenesis by preserving mitochondrial function, limiting reactive oxygen species (ROS) accumulation, and modulating injury-associated signaling pathways (Zhang et al., 2021). Our previous studies further suggested that SIRT3 exerts anti-ferroptotic and anti-fibrotic effects in renal resident cells, including tubular epithelial cells (Xiong et al., 2025; Cui et al., 2026). Given its central role in metabolic homeostasis and cellular protection, SIRT3 has emerged as a promising candidate target for CKD intervention.

In recent years, the concept of the gut–kidney axis has provided a new systems-level perspective for understanding the pathogenesis and progression of CKD (Tsuji et al., 2025). Increasing evidence indicates that CKD is not merely a localized renal disorder, but is also closely associated with gut microbiota dysbiosis, disruption of the intestinal mucosal barrier, and abnormal accumulation of gut-derived metabolites (Hayeeawaema et al., 2023). The uremic milieu can impair intestinal epithelial barrier integrity, facilitating the translocation of endotoxins and toxic metabolites into the circulation, which in turn promotes chronic low-grade inflammation and aggravates renal injury (Yang et al., 2024). Conversely, declining renal function can further reshape the intestinal microenvironment, thereby creating a vicious cycle. Although the local renoprotective role of SIRT3 has received considerable attention, whether SIRT3 also contributes to the maintenance of intestinal barrier homeostasis and regulation of the gut microbiota under CKD conditions remains largely unexplored (Qin et al., 2024). Given its central role in mitochondrial function, oxidative stress, and metabolic balance, clarifying the potential involvement of SIRT3 in the gut–kidney axis may provide new insight into the systemic mechanisms underlying CKD.

On the basis of these considerations, we hypothesized that the protective role of SIRT3 in CKD extends beyond the kidney itself and may also involve the maintenance of intestinal mucosal barrier homeostasis and regulation of the gut microbiota (Hayeeawaema et al., 2023; Yang et al., 2024). Loss of SIRT3 may exacerbate injury and fibrosis in renal resident cells while simultaneously promoting intestinal barrier dysfunction and microbial dysbiosis, thereby accelerating CKD progression. To test this hypothesis, we employed an integrated *in vivo* and *in vitro* strategy. *In vivo*, we established an adenine-induced CKD model in global SIRT3 knockout mice and systematically evaluated the effects of SIRT3 deficiency on renal injury, fibrosis, colonic histological alterations, intestinal barrier-related indices, and gut microbial composition. *In vitro*, tubular epithelial cells were treated with 3-TYP to examine its effects on TGF-β1-induced cellular injury and extracellular matrix deposition. This study aimed to explore the potential mechanisms by which SIRT3 contributes to CKD progression from the perspective of gut–kidney axis-related alterations and to provide experimental evidence supporting the further evaluation of SIRT3 as a potential therapeutic target for CKD.

## 2 Materials and methods

### 2.1 Animal model and experimental design

Male global SIRT3 knockout mice on a C57BL/6 background were obtained from Cyagen Biosciences (Suzhou, China) and bred in the Laboratory Animal Center of Wuhan Halic Biotechnology Co., Ltd. Wild-type C57BL/6 male mice aged 6 to 8 weeks and weighing 20 ± 2 g were purchased from the Hubei Provincial Center for Disease Control and Prevention (Wuhan, China). All animals were housed in a standard specific pathogen-free facility under controlled temperature and humidity, a fixed light–dark cycle, and identical cage, bedding, diet, and water conditions, and were allowed to acclimatize for 1 week before the experiment.

According to the approved protocol, 42 male mice were initially prepared for the study. After acclimatization, 36 mice were randomly selected using a random number table and allocated to the formal experiment, with 9 mice per group. The remaining 6 mice were retained as reserve animals and were not included in the formal experiment or statistical analysis. The 36 mice were assigned to four groups: a Control group, consisting of wild-type mice fed a standard diet; a CKD group, consisting of wild-type mice fed a diet containing 0.2% adenine by weight; a SIRT3^−/−^ group, consisting of SIRT3 knockout mice fed a standard diet; and a CKD + SIRT3^−/−^ group, consisting of SIRT3 knockout mice fed a diet containing 0.2% adenine by weight (Viñuela-Berni et al., 2025). The experimental diets were purchased from Wuhan Chunzhilong Laboratory Animal Co., Ltd. (Wuhan, China).

During the 6-week experimental period, body weight, food intake, and water intake were monitored continuously. At the end of the experiment, fresh fecal samples were collected and immediately stored at −80°C. The mice were then deeply anesthetized with isoflurane and euthanized by cervical dislocation, and blood, kidney, and colon samples were harvested. Blood samples were allowed to stand at room temperature for 30 min and were then centrifuged at 3,000 rpm for 10 min to obtain serum, which was stored at −80°C until analysis. Colon length was measured and gross tissue morphology was recorded. Kidney and colon tissues designated for histological analysis were fixed in 4% paraformaldehyde, whereas the remaining tissues were snap-frozen in liquid nitrogen and stored at −80°C for subsequent analyses. All animal procedures were performed in strict accordance with the guidelines of the Animal Care and Use Committee of Wuhan Halic Biotechnology Co., Ltd.

### 2.2 . Cell culture and *in vitro* treatment

Human proximal tubular epithelial cells (HK-2; Wuhan Servicebio Technology Co., Ltd., Wuhan, China) were cultured in DMEM/F12 medium (Thermo Fisher Scientific, Waltham, MA, USA) supplemented with 10% fetal bovine serum (Thermo Fisher Scientific, Waltham, MA, USA) at 37°C in a humidified incubator containing 5% CO_2_.

To establish an *in vitro* profibrotic model, cells were treated with transforming growth factor-β1 (TGF-β1) at 10 ng/mL for 0–48 h. In the inhibition experiments, cells were pretreated with the SIRT3 inhibitor 3-TYP (Beyotime Biotechnology, Shanghai, China; catalog no. Y148914; purity, 98%) at a final concentration of 50 μM for 2 h, followed by incubation with TGF-β1 for an additional 24 h (Zhang et al., 2024).

Cell viability was assessed using a Cell Counting Kit-8 (CCK-8; Wuhan Servicebio Technology Co., Ltd., Wuhan, China; catalog no. G4103). HK-2 cells were seeded in 96-well plates at a density of 5 × 10^3^ cells per well in 100 μL complete medium. After cell attachment, the cells were treated according to the experimental design. At the end of treatment, 10 μL of CCK-8 working solution was added to each well, and the plates were incubated at 37°C in the dark for 30–60 min. Absorbance was measured at 450 nm using a microplate reader, and relative cell viability was calculated after blank correction.

### 2.3 Detection of cell apoptosis by TUNEL staining and flow cytometry

DNA fragmentation and apoptosis-related changes were evaluated using a TUNEL apoptosis fluorescence detection kit (Wuhan Servicebio Technology Co., Ltd., Wuhan, China; catalog no. G1504-50T). Staining was performed according to the manufacturer's instructions, and images were captured under a fluorescence microscope.

To further quantify the apoptotic rate, flow cytometry was performed. HK-2 cells in logarithmic growth phase were seeded into 12-well plates at a density of 1 × 10^5^ cells/mL, with 1 mL per well, and cultured at 37°C in 5% CO_2_. On the following day, cells were treated according to the experimental design for 24 h. The culture supernatant was collected into centrifuge tubes. Cells were then digested with 0.5 mL trypsin for 1–3 min, and digestion was terminated with culture medium once the cells had detached. The detached cells were combined with the collected supernatant. After centrifugation at 1,000 rpm for 5 min, the supernatant was discarded and the cells were washed twice with PBS. The cell pellet was then stained using an Annexin V-FITC/PI apoptosis detection kit (Beyotime Biotechnology, Shanghai, China; catalog no. C1062M). Briefly, cells were resuspended in 195 μL Annexin V-FITC binding buffer, followed by the addition of 5 μL Annexin V-FITC and 10 μL propidium iodide (PI). After gentle mixing, the cells were incubated in the dark at room temperature for 15 min. Flow cytometric analysis was then performed using a flow cytometer (FongCyteC2080, Beijing Layergen Biotechnology Co., Ltd., Beijing, China). Total apoptosis was defined as the sum of early apoptotic and late apoptotic cells.

### 2.4 Biochemical assays and ELISA

creatinine and urea levels were measured using colorimetric assay kits according to the manufacturers' instructions. In addition, serum concentrations of inflammatory and kidney injury-related markers were quantified using enzyme-linked immunosorbent assay (ELISA) kits, including tumor necrosis factor-α (TNF-α; Wuhan Servicebio Technology Co., Ltd., Wuhan, China; catalog no. GEM0004-48T), interleukin-6 (IL-6; Wuhan Servicebio Technology Co., Ltd., Wuhan, China; catalog no. GEM0001-48T), and neutrophil gelatinase-associated lipocalin (NGAL; Beyotime Biotechnology, Shanghai, China; catalog no. PN757). All assays were performed strictly in accordance with the manufacturers' protocols, and concentrations were calculated on the basis of the corresponding standard curves.

### 2.5 Histopathological staining and semiquantitative evaluation

Kidney and colon tissues were fixed in 4% paraformaldehyde for 24 h, dehydrated through a graded ethanol series, embedded in paraffin, and sectioned at a thickness of 6 μm. After deparaffinization, sections were stained with hematoxylin and eosin (H&E) to assess overall histopathological injury. For kidney tissues, a periodic acid-Schiff (PAS) staining kit (Beijing Solarbio Science & Technology Co., Ltd., Beijing, China) was used to evaluate structural renal injury, including alterations of the tubular brush border, tubular dilatation, and cast formation. A Masson's trichrome staining kit (Xi'an Haotian Bioengineering Co., Ltd., Xi'an, China) was used to assess collagen deposition and the extent of renal interstitial fibrosis. For colon tissues, an Alcian blue (AB) staining kit (Hubei Servicebio Technology Co., Ltd., Wuhan, China) was used to detect mucus layer-associated mucin expression and goblet cell distribution. All stained sections were examined and photographed under a light microscope.

Histological injury scoring was independently performed by two investigators in a blinded manner. Renal injury was evaluated mainly on the basis of PAS staining. For each section, five randomly selected non-overlapping high-power fields ( × 200) were scored, and the mean value was used for statistical analysis. The relative fibrotic area in Masson-stained sections and the percentages of PAS- and AB-positive areas were quantified using ImageJ software.

### 2.6 Western blot analysis

Total protein was extracted from kidney tissue, colon tissue, and cultured cells using RIPA lysis buffer (Beyotime Biotechnology, Shanghai, China) supplemented with a protease inhibitor cocktail (Merck, Darmstadt, Germany). Protein concentrations were determined using a BCA protein assay kit (Beyotime Biotechnology, Shanghai, China). Equal amounts of protein were separated by sodium dodecyl sulfate-polyacrylamide gel electrophoresis (SDS-PAGE) and transferred onto polyvinylidene fluoride (PVDF) membranes. Membranes were blocked with 5% non-fat milk at room temperature for 1 h and then incubated overnight at 4°C with the appropriate primary antibodies.

The following primary antibodies were used: anti-β-actin antibody (Servicebio, Wuhan, China; catalog no. GB15003; 1:5000), anti-SIRT3 antibody (Servicebio, Wuhan, China; catalog no. GB115590-100; 1:1000), anti-α-SMA antibody (Servicebio, Wuhan, China; catalog no. GB111364-100; 1:1000), anti-Collagen I antibody (Servicebio, Wuhan, China; catalog no. GB11022-100; 1:1000), anti-Fibronectin antibody (Servicebio, Wuhan, China; catalog no. GB114491-100; 1:1000), anti-ZO-1 antibody (Servicebio, Wuhan, China; catalog no. GB111402-100; 1:1000), and anti-Occludin antibody (Servicebio, Wuhan, China; catalog no. GB111401-100; 1:1000). Among these, SIRT3, α-SMA, Collagen I, and Fibronectin were mainly used for kidney tissues and cell samples, whereas ZO-1 and Occludin were mainly used to detect intestinal barrier-related proteins in colon tissues. After washing, membranes were incubated with HRP-conjugated goat anti-rabbit IgG secondary antibody (Servicebio, Wuhan, China; catalog no. GB23303; 1:3000) at room temperature for 1.5 h. Band intensities were quantified using ImageJ software after background subtraction. For each sample, the integrated density of the target protein band was first normalized to the corresponding internal control band from the same lane, namely β-actin or GAPDH, as indicated in each figure. The normalized values were then divided by the mean value of the Control group, which was set as 1.0, to obtain relative protein expression levels.

### 2.7 16S rRNA gene sequencing and gut microbiota analysis

Total genomic DNA was extracted from fecal samples strictly according to the kit instructions. DNA integrity was assessed by 1% agarose gel electrophoresis, and DNA concentration and purity were measured using a NanoDrop 2000 and Qubit 4.0. The V3–V4 hypervariable region of the bacterial 16S rRNA gene was amplified by PCR using barcode-tagged specific primers and TransStart FastPfu high-fidelity DNA polymerase. PCR products were purified using Agencourt AMPure XP beads, and sequencing libraries were constructed and subjected to paired-end high-throughput sequencing on an Illumina platform (Bharti and Grimm, 2019).

Raw sequencing data were subjected to quality control before downstream analysis. Low-quality reads and reads containing ambiguous bases were filtered using Trimmomatic (version 0.36). Paired-end reads were then merged using FLASH (version 1.2.0), and chimeric sequences were removed using Vsearch (version 2.7.1) to obtain high-quality effective sequences. Operational taxonomic unit (OTU) clustering was performed on the QIIME 2 platform using a 97% sequence similarity threshold, and taxonomic annotation was performed based on the SILVA database.

Alpha diversity was evaluated using the Shannon index. Beta diversity was analyzed on the basis of the Bray-Curtis distance matrix and visualized by non-metric multidimensional scaling (NMDS) and principal coordinates analysis (PCoA). Differential taxa among groups were identified using linear discriminant analysis effect size (LEfSe). Briefly, taxa with significantly different abundances among groups were first screened using the Kruskal–Wallis rank-sum test, followed by pairwise comparisons using the Wilcoxon rank-sum test. The effect size of differential taxa was then evaluated by linear discriminant analysis (LDA). The significance criteria were set at *P* < 0.05 and LDA > 2.0 (Nearing et al., 2022).

### 2.8 Statistical analysis

All statistical analyses and data visualization were performed using GraphPad Prism 8.0 (GraphPad Software, San Diego, CA, USA) and R software. Quantitative data are presented as mean ± standard deviation (SD). For comparisons among multiple groups, one-way analysis of variance (ANOVA) followed by Tukey's *post hoc* test was used for data with normal distribution and homogeneity of variance, whereas non-parametric tests were applied when these assumptions were not met. Repeated-measures data, including body weight, food intake, and water intake, were analyzed using two-way repeated-measures ANOVA. Spearman's rank correlation analysis was used to evaluate the associations between key gut microbial taxa and renal pathological or functional indicators. All *in vitro* experiments were independently repeated at least three times. A *P* value < 0.05 was considered statistically significant.

## 3 Results

### 3.1 SIRT3 deficiency aggravates adenine-induced renal dysfunction and inflammatory responses in mice

**Figure 1:**
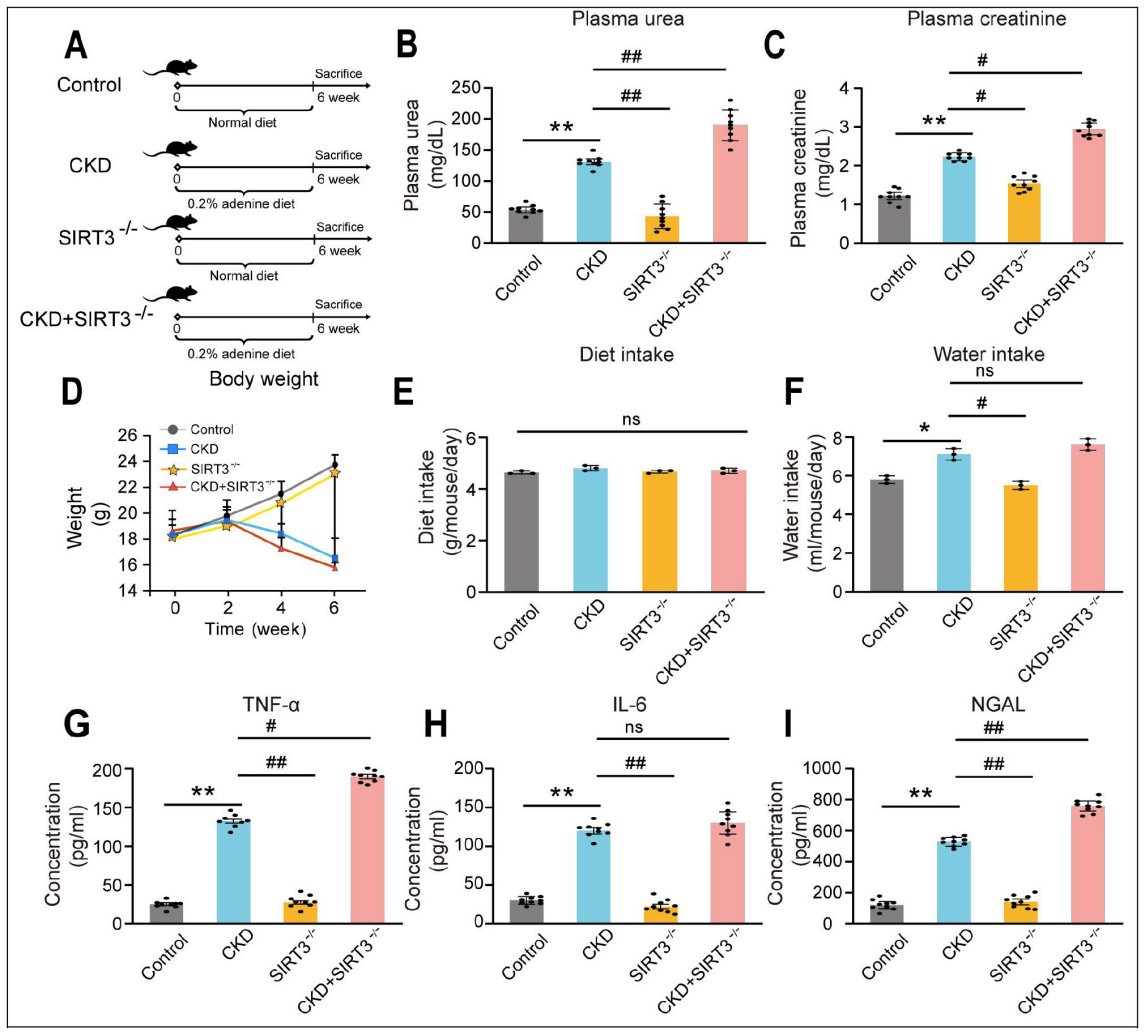
SIRT3 deficiency aggravates adenine-induced renal dysfunction and inflammatory responses in mice. **(A)** Experimental design and grouping of the animal study. **(B)** urea levels in the Control, CKD, SIRT3^−/−^ and CKD + SIRT3^−/−^ groups. **(C)** creatinine levels in each group. **(D)** Dynamic changes in body weight during the experimental period. **(E)** Food intake in each group,Each dot represents one cage mean value, normalized to the number of mice per cage (*n* = 3 cages/group). **(F)** Water intake in each group. **(G)** Serum TNF-α levels in each group. **(H)** Serum IL-6 levels in each group. **(I)** Serum NGAL levels in each group. Data are presented as mean ± SD. ^*^P < 0.05 *and* ^**^P < 0.01 vs. Control; ^#^*P* < 0.05 and ^*##*^*P* < 0.01 vs. CKD; *P* < 0.05 was considered statistically significant. ns, not significant.

To investigate the role of SIRT3 in CKD, an adenine-induced CKD mouse model was established, and four groups were included for comparison: Control, CKD, SIRT3^−/−^, and CKD + SIRT3^−/−^ (Figure 1A). Compared with the Control group, the SIRT3^−/−^ group alone showed no obvious abnormalities in urea, creatinine, or inflammation-related indices. In contrast, urea and creatinine levels were markedly elevated in the CKD group, indicating impaired renal function (Figures 1B, C). These abnormalities were further aggravated in the CKD + SIRT3^−/−^ group, suggesting that SIRT3 deficiency exacerbated renal dysfunction under CKD conditions (Figures 1B, C).

Dynamic monitoring showed that the SIRT3^−/−^ group alone exhibited a trend similar to that of the Control group, whereas mice in the CKD and CKD + SIRT3^−/−^ groups developed a more evident decline in body weight during the later stage of modeling (Figure 1D). Food intake did not differ substantially among groups, whereas water intake increased in the CKD group and further increased in the CKD + SIRT3^−/−^ group (Figures 1E, F). Further analysis of systemic inflammatory and kidney injury-related markers showed that serum TNF-α, IL-6, and NGAL levels were significantly elevated in the CKD group compared with the Control group (Figures 1G–I). Among these markers, TNF-α and NGAL were more pronounced in the CKD + SIRT3^−/−^ group, whereas IL-6 showed no significant additional increase compared with the CKD group (Figures 1G–I). Collectively, these findings indicate that SIRT3 deficiency does not cause overt renal dysfunction under basal conditions but markedly aggravates adenine-induced renal injury and inflammatory responses in CKD.

### 3.2 SIRT3 deficiency exacerbates renal histopathological injury and fibrosis in CKD mice

**Figure 2:**
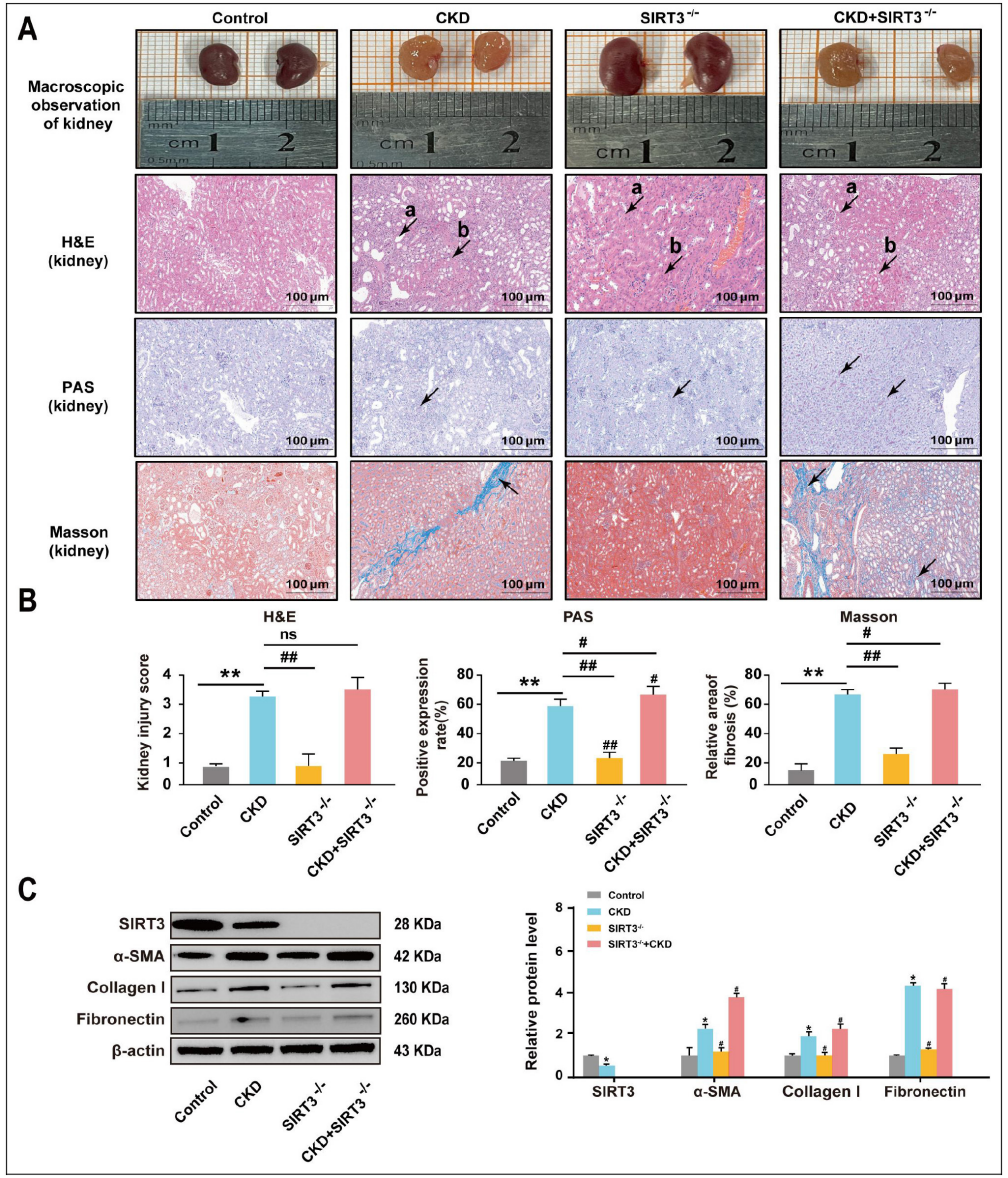
SIRT3 deficiency exacerbates renal histopathological injury and fibrosis in CKD mice. **(A)** Representative gross images of kidneys and representative hematoxylin and eosin, periodic acid-Schiff, and Masson's trichrome staining images from the Control, CKD, SIRT3^−/−^ and CKD + SIRT3^−/−^ groups. **(B)** Quantitative analysis of renal histopathological injury and relative fibrotic area. **(C)** Western blot analysis and quantification of SIRT3, α-SMA, Collagen I, and Fibronectin expression in kidney tissues. Scale bars are shown in the panels. Data are presented as mean ± SD. ^*^P < 0.05 *and* ^**^P < 0.01 vs. Control; ^#^*P* < 0.05 and ^*##*^*P* < 0.01 vs. CKD; *P* < 0.05 was considered statistically significant. ns, not significant.

To further evaluate the impact of SIRT3 deficiency on renal pathological injury, gross morphology, histological staining, and fibrosis-related protein expression were examined in kidney tissues from each group (Figures 2A–C). The SIRT3^−/−^ group alone showed gross and histological features largely comparable to those of the Control group, without obvious pathological injury resembling that observed in the CKD group (Figures 2A, B). By contrast, clear morphological alterations were observed in the kidneys of CKD mice. H&E staining revealed tubular dilatation, disorganization of the tubular lumen, and interstitial injury, whereas PAS staining demonstrated marked tubular structural damage (Figures 2A, B). Masson's trichrome staining further showed substantial collagen deposition in the renal interstitium (Figures 2A, B). These pathological changes were more severe in the CKD + SIRT3^−/−^ group, indicating that SIRT3 deficiency further aggravated renal injury and fibrosis in the CKD setting (Figures 2A, B).

Western blot analysis showed that SIRT3 expression was decreased in the CKD group compared with the Control group, whereas α-SMA, Collagen I, and Fibronectin were markedly upregulated (Figure 2C). The expression of these fibrosis-related proteins was further increased in the CKD + SIRT3^−/−^ group (Figure 2C). Together, these findings indicate that SIRT3 deficiency alone does not induce overt renal pathological abnormalities but significantly accelerates structural kidney injury and fibrotic progression under CKD conditions.

### 3.3 SIRT3 deficiency aggravates colonic injury and is associated with intestinal mucosal barrier impairment in CKD mice

**Figure 3:**
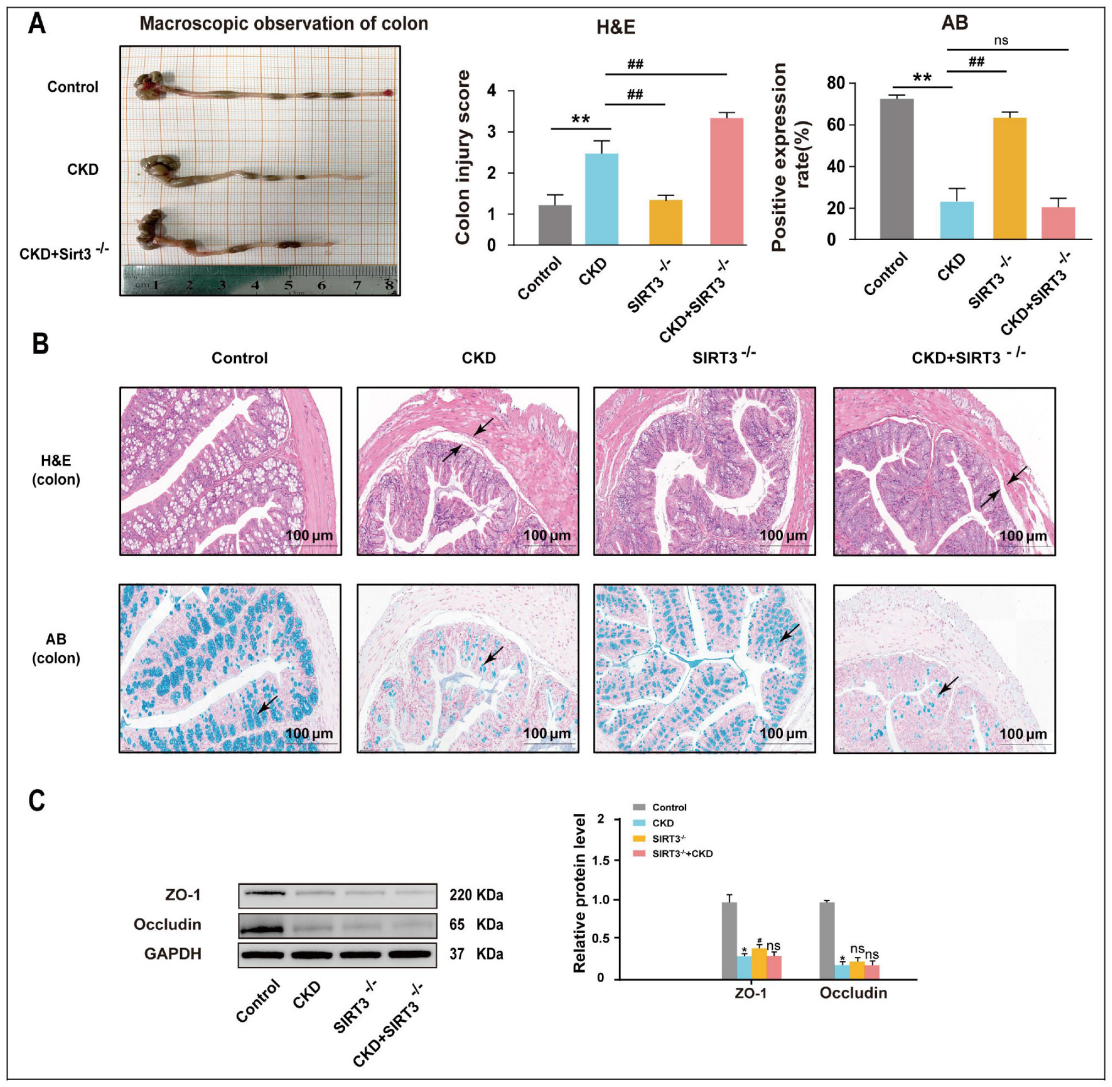
SIRT3 deficiency aggravates colonic injury and impairs the intestinal mucosal barrier in CKD mice. **(A)** Representative gross images of the colon and representative hematoxylin and eosin and Alcian blue staining images from the Control, CKD, SIRT3^−/−^ and CKD + SIRT3^−/−^ groups. **(B)** Quantitative analysis of colonic injury and AB-positive area. **(C)** Western blot analysis and quantification of ZO-1 and Occludin expression in colon tissues. Scale bars are shown in the panels. Data are presented as mean ± SD. ^*^P < 0.05 *and* ^**^P < 0.01 vs. Control; ^#^*P* < 0.05 and ^*##*^*P* < 0.01 vs. CKD; *P* < 0.05 was considered statistically significant.

To determine whether SIRT3 deficiency was associated with aggravated intestinal injury, colonic tissues were further examined (Figures 3A–C). The SIRT3^−/−^ group alone showed colon length and histological appearance broadly comparable to those of the Control group, without obvious colonic pathological abnormalities. In contrast, the CKD group exhibited shortened colons, and H&E staining revealed epithelial disorganization, mucosal injury, and inflammatory changes in the colonic mucosa. AB staining showed a marked reduction in mucus-associated positive staining, suggesting impaired goblet cell function and mucus barrier integrity. In the CKD + SIRT3^−/−^ group, the colonic injury score was further increased, whereas the AB-positive area remained reduced without a significant difference compared with the CKD group (Figures 3A, B).

Western blot analysis of mucosal barrier-related proteins showed that ZO-1 and Occludin expression was significantly decreased in the CKD group compared with the Control group (Figure 3C). After re-quantification of the Western blot bands, ZO-1 and Occludin remained at low levels in the CKD + SIRT3^−/−^ group; however, their levels were not significantly different from those in the CKD group (Figure 3C). These findings indicate that SIRT3 deficiency further aggravates CKD-associated colonic histological injury and mucus barrier impairment, while tight junction proteins are markedly reduced under CKD conditions and remain suppressed in CKD + SIRT3^−/−^ mice.

### 3.4 Inhibition of SIRT3 promotes TGF-β1-induced profibrotic phenotypes and apoptosis in HK-2 cells

**Figure 4:**
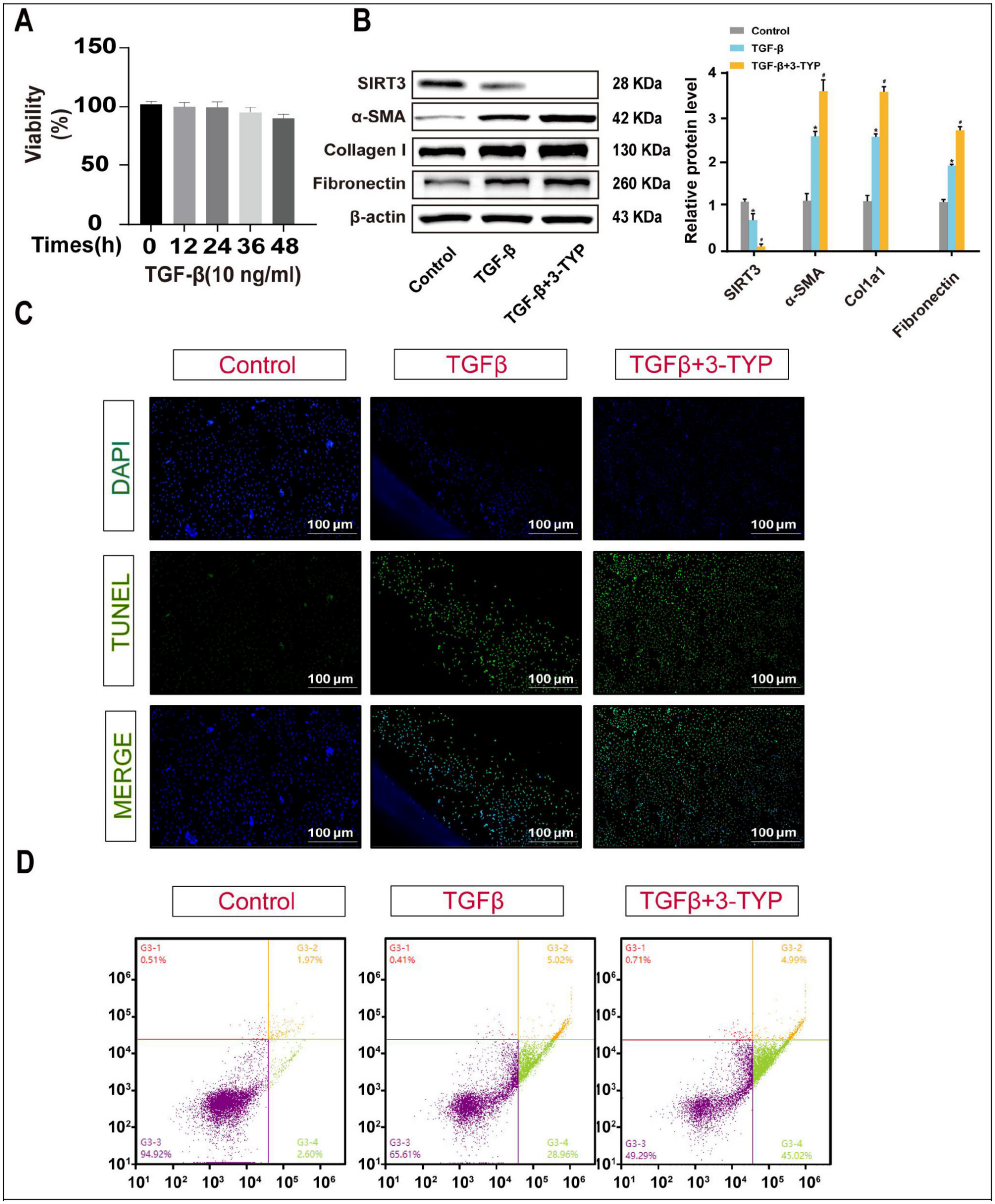
Inhibition of SIRT3 enhances TGF-β1-induced profibrotic responses and apoptosis in HK-2 cells. **(A)** CCK-8 analysis of HK-2 cell viability after treatment with 10 ng/mL TGF-β1 for the indicated durations. **(B)** Western blot analysis and quantification of SIRT3, α-SMA, Collagen I, and Fibronectin expression in HK-2 cells treated with TGF-β1 in the presence or absence of 3-TYP. **(C)** Representative TUNEL staining images showing apoptotic HK-2 cells in each group. **(D)** Flow cytometric analysis and quantification of apoptotic rates in HK-2 cells. Data are presented as mean ± SD. ^*^P < 0.05 and ^**^P < 0.01 vs. Control; ^#^*P* < 0.05 and ^*##*^*P* < 0.01 vs. TGF-β1; *P* < 0.05 was considered statistically significant.

To further validate the regulatory role of SIRT3 in tubular epithelial injury at the *in vitro* level, HK-2 cells were exposed to TGF-β1 to establish a profibrotic model, and SIRT3 activity was inhibited with 3-TYP. CCK-8 analysis showed that treatment with 10 ng/mL TGF-β1 for 0–48 h did not cause an obvious overall reduction in cell viability, indicating that this condition was suitable for subsequent mechanistic studies (Figure 4A).

Western blot analysis demonstrated that TGF-β1 treatment reduced SIRT3 expression in HK-2 cells, while α-SMA, Collagen I, and Fibronectin were markedly upregulated (Figure 4B). Compared with the TGF-β1-only group, pretreatment with 3-TYP further increased the expression of these profibrotic proteins (Figure 4B). TUNEL staining showed that TGF-β1 induced apoptosis in HK-2 cells, and the TUNEL-positive signal was further enhanced by combined 3-TYP treatment (Figure 4C). Flow cytometry likewise showed that the apoptotic rate increased in the TGF-β1 group and was further elevated in the TGF-β1 + 3-TYP group (Figure 4D). These results indicate that pharmacological inhibition of SIRT3 enhances TGF-β1-induced cellular injury, profibrotic responses, and apoptosis in HK-2 cells.

### 3.5 SIRT3 deficiency under CKD conditions is associated with further alterations in gut microbial diversity and community structure

**Figure 5:**
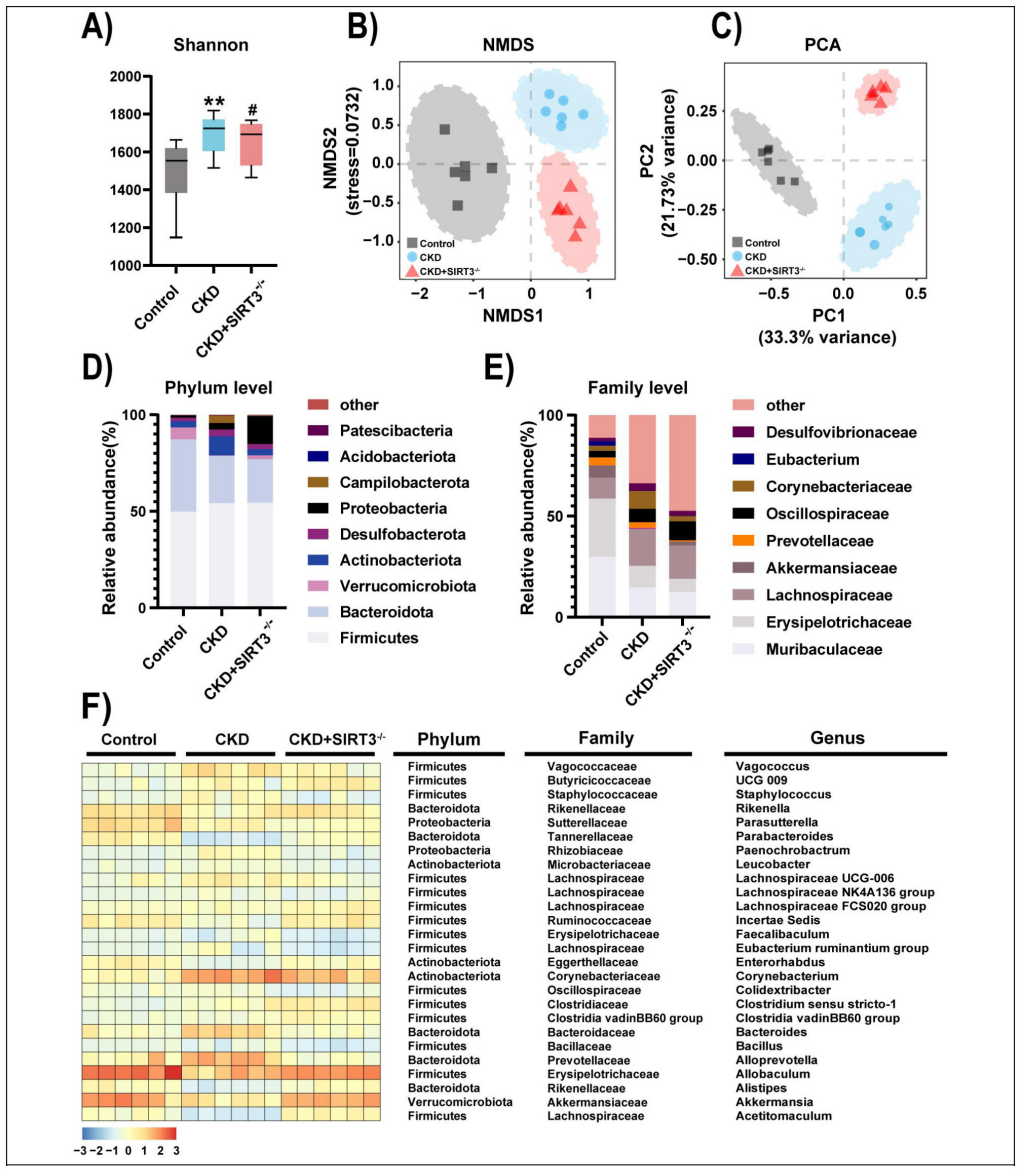
SIRT3 deficiency under CKD conditions is associated with altered gut microbial diversity and community structure. **(A)** Alpha diversity analysis based on the Shannon index in the Control, CKD, and CKD + SIRT3^−/−^ groups. **(B)** Non-metric multidimensional scaling analysis of gut microbial community structure. **(C)** Principal coordinates analysis of gut microbial community structure. **(D)** Relative abundance of major bacterial taxa at the phylum level. **(E)** Relative abundance of dominant bacterial taxa at the family level. **(F)** Heatmap showing differential genus-level abundance patterns among groups. **P < 0.01 vs. Control; ^#^*P* < 0.05 vs. CKD.

To further evaluate the relationship between SIRT3 deficiency and gut microbial alterations under CKD conditions, 16S rRNA gene sequencing was performed on fecal samples from the Control, CKD, and CKD + SIRT3^−/−^ groups. Alpha diversity analysis showed significant differences in the Shannon index among the three groups, and the CKD + SIRT3^−/−^ group displayed a diversity pattern distinct from that of the CKD group (Figure 5A). Beta diversity analysis showed relatively clear clustering separation among the three groups in both NMDS and PCoA plots, indicating marked remodeling of the overall gut microbial community structure under CKD conditions and in the presence of SIRT3 deficiency (Figures 5B, C).

At the phylum level, Firmicutes and Bacteroidota remained the dominant taxa in all groups, but their relative abundance profiles shifted, accompanied by fluctuations in Verrucomicrobiota, Actinobacteriota, Desulfobacterota, and Proteobacteria (Figure 5D). At the family level, dominant taxa including Muribaculaceae, Erysipelotrichaceae, Lachnospiraceae, Akkermansiaceae, Prevotellaceae, and Oscillospiraceae also showed marked differences among groups (Figure 5E). Heatmap analysis at the genus level further revealed distinct abundance patterns for multiple genera across groups, suggesting that SIRT3 deficiency under CKD conditions is associated with more pronounced gut microbial dysbiosis (Figure 5F).

### 3.6 LEfSe analysis identifies characteristic microbial signatures associated with SIRT3 deficiency under CKD conditions

**Figure 6:**
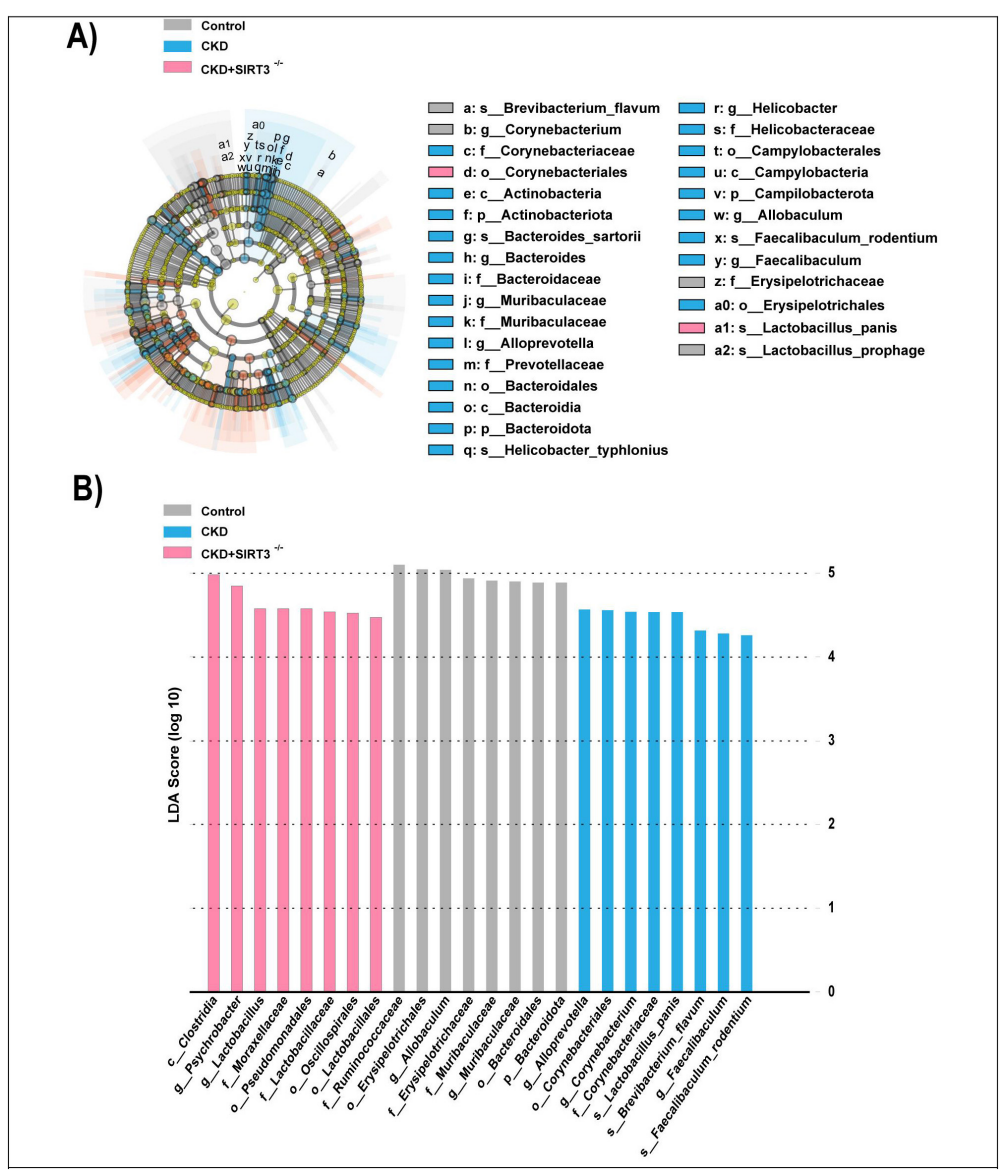
LEfSe analysis identifies characteristic microbial signatures associated with SIRT3 deficiency under CKD conditions. **(A)** Cladogram showing the phylogenetic distribution of differentially enriched taxa among the Control, CKD, and CKD + SIRT3^−/−^ groups. **(B)** Linear discriminant analysis scores of differentially abundant taxa identified by linear discriminant analysis effect size.

To further identify discriminative microbial taxa among groups, LEfSe analysis was performed on the three groups of samples. The phylogenetic cladogram showed that each group was characterized by its own enriched microbial branches (Figure 6A). LDA results showed that the Control group was mainly enriched in Erysipelotrichaceae, Erysipelotrichales, Allobaculum, Muribaculaceae, Bacteroidales, Bacteroidia, and Bacteroidota (Figure 6B). The CKD group was mainly enriched in Alloprevotella, Corynebacteriales, Corynebacterium, Corynebacteriaceae, Lactobacillus panis, Brevibacterium flavum, Faecalibaculum, and Faecalibaculum rodentium (Figure 6B). In contrast, the CKD + SIRT3^−/−^ group was mainly enriched in Clostridia, Psychrobacter, Lactobacillus, Moraxellaceae, Pseudomonadales, Lactobacillaceae, Oscillospirales, and Lactobacillales (Figure 6B). These findings indicate that SIRT3 deficiency under CKD conditions is associated with a characteristic set of differential microbial signatures.

### 3.7 Specific gut microbial taxa are significantly associated with renal fibrosis, renal function, and general physiological indices

**Figure 7:**
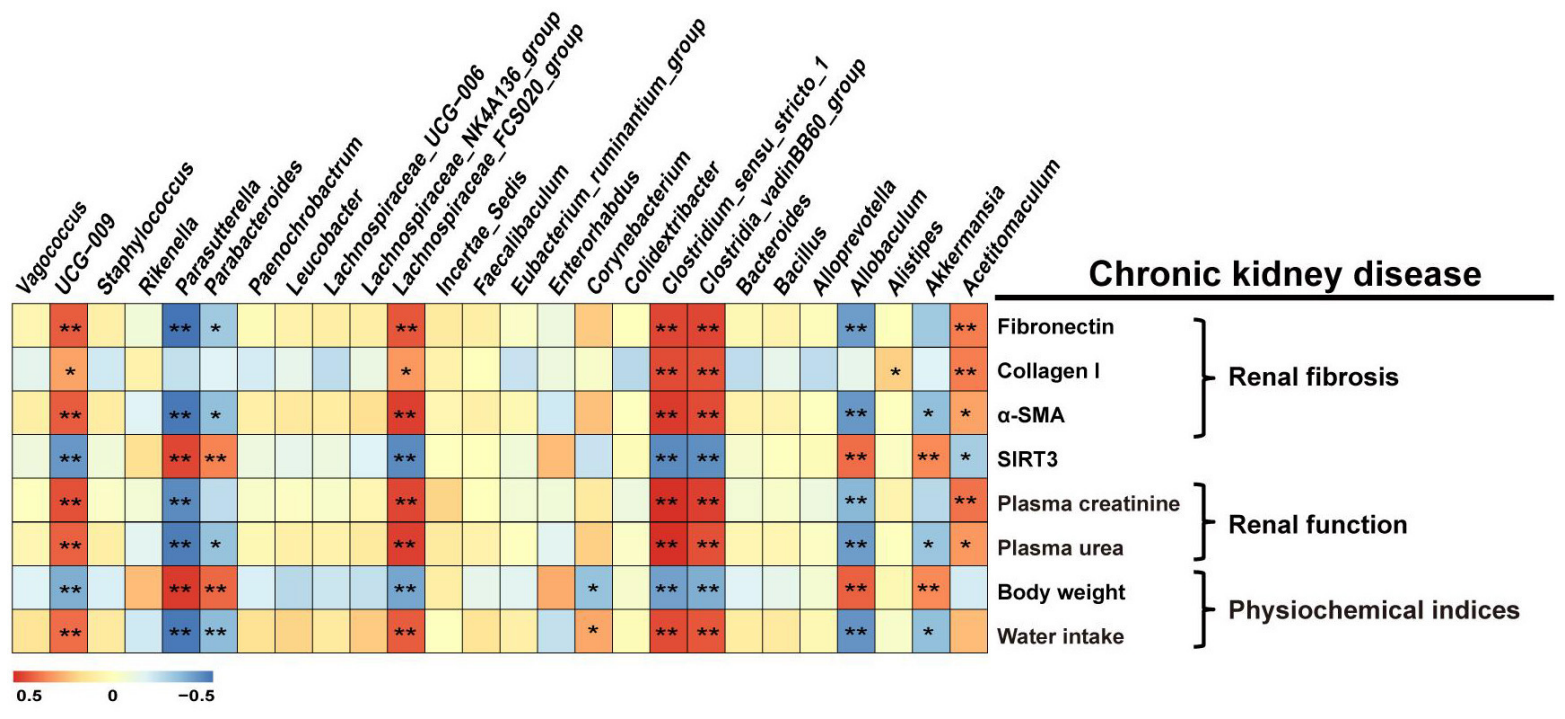
Correlations between specific gut microbial taxa and indices of renal fibrosis and renal dysfunction. Spearman correlation analysis showing the associations between key differential microbial taxa and renal pathological or functional parameters, including Fibronectin, Collagen I, α-SMA, creatinine, urea, body weight, and water intake. Positive correlations are shown in red and negative correlations are shown in blue, with color intensity reflecting correlation strength.

To explore the relationship between key differential taxa and disease phenotypes, Spearman correlation analysis was further performed (Figure 7). The results showed that the abundance of multiple microbial taxa was significantly correlated with renal fibrosis, renal function, and general physiological status. Specifically, Vagococcus, UCG-009, Lachnospiraceae UCG-006, Lachnospiraceae NK4A136 group, Corynebacterium, Colidextribacter, Clostridium sensu stricto-1, and the Clostridia vadinBB60 group were generally positively correlated with Fibronectin, Collagen I, α-SMA, creatinine, urea, and water intake, while showing a negative correlation with body weight (Figure 7). In contrast, Parasutterella, Parabacteroides, Alloprevotella, Allobaculum, and Akkermansia were generally negatively correlated with multiple indices of renal injury and fibrosis (Figure 7). These findings suggest that gut microbial alterations associated with SIRT3 deficiency under CKD conditions are significantly linked to renal pathological injury and functional abnormalities.

## 4 Discussion

Chronic kidney disease is a progressive clinical syndrome characterized by a sustained decline in renal function, persistent activation of inflammation, tubular injury, and progressive tubulointerstitial fibrosis (Francis et al., 2024; Mark et al., 2025). Inflammation and fibrosis are not only major drivers of disease progression but also the pathological basis for irreversible structural and functional renal damage (Qin et al., 2024; Miguel et al., 2025). As the disease advances, normal renal parenchyma is gradually replaced by excessive extracellular matrix deposition, ultimately leading to end-stage kidney disease. For this reason, further elucidation of the mechanisms underlying inflammation and fibrosis in CKD, as well as the identification of novel therapeutic targets, remains highly important. Against this background, the present study focused on SIRT3 and systematically evaluated its role in CKD at both the *in vivo* and *in vitro* levels. *In vivo*, we examined the effects of SIRT3 deficiency on renal functional indices, inflammatory responses, renal fibrosis, colonic mucosal barrier integrity, and gut microbial alterations. *In vitro*, we used TGF-β1-induced HK-2 cell injury to further assess the role of SIRT3 in profibrotic responses and apoptosis in tubular epithelial cells. Overall, our findings indicate that SIRT3 deficiency amplifies inflammation, fibrosis, and barrier injury under CKD conditions. These data further suggest that SIRT3 may serve as an important regulatory link between local renal injury and gut-kidney axis dysfunction.

In this study, CKD was induced in mice using a 0.2% adenine-containing diet, which reliably reproduced typical pathological features, including deterioration in general condition, renal dysfunction, tubular injury, and tubulointerstitial fibrosis. Previous studies have shown that the adenine model is primarily driven by the formation and intratubular deposition of 2,8-dihydroxyadenine crystals, which trigger tubular obstruction, interstitial inflammation, collagen deposition, and chronic fibrotic progression (Yang et al., 2024). This model has also been reported to recapitulate not only renal dysfunction but also inflammatory activation, oxidative stress, and intestinal impairment, making it well suited for studying progressive CKD and gut-kidney axis abnormalities. In this respect, the animal model used in the present study is broadly consistent with previous reports and provides a reliable platform for evaluating the impact of SIRT3 deficiency on disease aggravation.

The kidney is one of the most metabolically active organs in the body. Its high energy demand is closely related to continuous blood filtration, active reabsorption of solutes and water, maintenance of electrolyte and acid-base balance, and regulation of blood pressure (Azzouz and Yan, 2025). The proximal tubule is responsible for the majority of filtrate reabsorption and is therefore highly enriched in mitochondria, making it particularly vulnerable to disturbances in energy metabolism (Peng et al., 2024). Podocytes likewise require substantial energy to maintain cytoskeletal integrity and preserve the glomerular filtration barrier. In this context, the sirtuin family, as an important sensor of cellular metabolic status, plays a critical role in renal homeostasis. Among these proteins, SIRT3 is predominantly localized in mitochondria and is closely associated with the maintenance of tubular energy homeostasis and antioxidant defense. Previous studies further suggest that SIRT3 participates in regulating mitochondrial dynamics and functional mitochondrial trafficking in renal cells, thereby supporting mitochondrial network stability, sustained energy production, and cellular resistance to oxidative stress (Zhang et al., 2021). SIRT3 may therefore represent an important molecular link between the high metabolic demands of the kidney, mitochondrial homeostasis, and susceptibility to injury.

Under pathological conditions, renal fibrosis is a nearly universal outcome of progressive CKD and represents the key basis for irreversible deterioration of renal structure and function. Previous studies have shown that members of the sirtuin family, particularly SIRT1 and SIRT3, play important protective roles against renal fibrosis (Chou et al., 2021). In UUO and 5/6 nephrectomy models, SIRT1 has been shown to be inversely associated with TGF-β signaling activity, and enhancement of SIRT1 attenuates renal inflammation and reduces the accumulation of type IV collagen, fibronectin, and MMP-7 through deacetylation of Smad3 and Smad4, thereby slowing the progression of tubulointerstitial fibrosis (He et al., 2010; Tan et al., 2025). Likewise, SIRT3 has also been reported to exert protective effects in models of renal injury and fibrosis, whereas its deficiency increases susceptibility to oxidative stress, promotes apoptosis, and aggravates renal dysfunction and interstitial fibrosis. Our findings are broadly consistent with these observations. In adenine-induced CKD mice, we observed increased urea, creatinine, TNF-α, IL-6, and NGAL levels, accompanied by marked tubular structural damage, enhanced collagen deposition, and upregulation of α-SMA, Collagen I, and Fibronectin. These alterations were further aggravated in the setting of SIRT3 deficiency. *In vitro*, TGF-β1 treatment decreased SIRT3 expression in HK-2 cells, and pharmacological inhibition of SIRT3 with 3-TYP further enhanced profibrotic protein expression and apoptosis. Together, these findings indicate that both adenine and TGF-β1 promote CKD-related pathological progression by inducing inflammation, fibrosis, and cell death, whereas SIRT3 deficiency further amplifies this process.

Previous studies have provided substantial evidence that gut microbiota dysbiosis and disruption of the intestinal mucosal barrier are important components of CKD progression (Pei et al., 2023; Liu W. et al., 2025). The intestinal barrier, consisting of the mucus layer, epithelial cells, and tight junction proteins, serves as a critical interface that restricts the translocation of luminal bacteria, endotoxins, and gut-derived toxic metabolites into the systemic circulation (Vaziri et al., 2013; Meijers et al., 2018). Under CKD conditions, the uremic milieu and gut microbiota dysbiosis can disrupt epithelial tight junctions and increase intestinal permeability, thereby facilitating the entry of microbial products and noxious metabolites into the circulation (Vaziri, 2012; Krukowski et al., 2023). These events may promote systemic inflammation, oxidative stress, and immune activation, ultimately contributing to tubular injury and renal fibrotic progression. Therefore, intestinal barrier dysfunction is not merely a secondary manifestation of CKD, but may function as an active amplifier of gut-kidney axis injury (Krukowski et al., 2026).

Investigations in adenine-induced CKD models have shown not only stable renal injury but also marked gastrointestinal dysfunction and barrier impairment (Yang et al., 2025). Interventions targeting the gut-kidney axis have been reported to reduce uremic toxin levels and improve renal function, suggesting that alterations in the intestinal microenvironment are not merely secondary phenomena but active contributors to CKD pathogenesis (Pei et al., 2023). More recent studies have further demonstrated that lactulose- or *Akkermansia muciniphila*-based interventions can improve intestinal barrier function and renal phenotypes in adenine-associated CKD, thereby supporting the therapeutic relevance of the gut microbiota in this setting. In contrast to these studies, the present work did not simply describe CKD-associated microbial dysbiosis (Fu et al., 2025), but further incorporated SIRT3 into this pathological framework. Our results showed that, under adenine-induced CKD conditions, SIRT3 deficiency not only aggravated renal dysfunction, inflammatory responses, and tubulointerstitial fibrosis, but was also accompanied by more severe colonic injury, downregulation of tight junction proteins, and greater abnormalities in gut microbial diversity and community structure. These findings are consistent with previous work in supporting a close association between intestinal microenvironmental abnormalities and CKD progression. However, unlike earlier studies that mainly emphasized CKD-driven microbial dysbiosis or the protective effects of microbiota-based interventions, our findings suggest that SIRT3 deficiency may represent an upstream factor that amplifies CKD-associated barrier disruption and microbial imbalance. The relationship between SIRT3 and gut microbiota may be bidirectional rather than strictly unidirectional. On the one hand, SIRT3 may influence the intestinal microenvironment by regulating mitochondrial homeostasis, oxidative stress, epithelial energy metabolism, and inflammatory responses. Recent evidence indicates that SIRT3 can protect intestinal barrier integrity and attenuate colonic inflammation and oxidative stress, suggesting that loss of SIRT3 may weaken epithelial resistance to CKD-associated intestinal injury and thereby favor microbial dysbiosis. On the other hand, gut microbiota and microbiota-derived metabolites may reciprocally regulate SIRT3-related pathways. Because SIRT3 activity depends on NAD+ availability, microbial metabolites that affect host NAD+ metabolism, mitochondrial function, redox balance, and inflammatory signaling may potentially modulate SIRT3 expression or activity (Niño-Narvión et al., 2023). Therefore, our findings support a possible SIRT3–intestinal barrier–gut microbiota axis in CKD, but the direction and causality of this interaction remain to be determined (Zhra et al., 2025).

Previous studies have shown that CKD is often accompanied by characteristic remodeling of the gut microbiota, and that such changes are closely associated with amplified inflammation and worsening renal function (Lazarevic et al., 2024). Clinical studies have reported increases in taxa such as Lactobacillus, Clostridium sensu stricto, and *Alloprevotella*, along with relative reductions in *Akkermansia* and *Parasutterella* in patients with CKD, and some of these taxa correlate with disease severity. *Akkermansia*-related interventions have also been shown to improve intestinal barrier function and alleviate renal injury in CKD models (Lambert et al., 2023). Our findings are generally consistent with this pattern. In the present study, *Allobaculum* and *Muribaculaceae* were mainly enriched in the Control group, *Alloprevotella*, Corynebacterium, and *Faecalibaculum* were enriched in the CKD group, and the CKD + SIRT3^−/−^ group showed further enrichment of Clostridia, Lactobacillus, and *Oscillospirales*. Correlation analysis further revealed that Clostridium sensu stricto-1, Corynebacterium, and related taxa were generally positively associated with Fibronectin, Collagen I, α-SMA, creatinine, and urea, whereas *Akkermansia, Parasutterella, Allobaculum*, and Parabacteroides showed overall negative associations with multiple indices of renal injury. These association patterns may have biological relevance and deserve further interpretation. The taxa positively associated with renal dysfunction and fibrotic markers, including Clostridium sensu stricto-1, Corynebacterium, Lachnospiraceae-related taxa, Colidextribacter, and the Clostridia vadinBB60 group, may represent a dysbiotic microbial configuration that emerges under the uremic and inflammatory milieu of CKD. In this context, their positive correlations with creatinine, urea, Fibronectin, Collagen I, and α-SMA suggest that these taxa may be linked to aggravated renal functional decline and extracellular matrix remodeling. This does not necessarily indicate that each taxon directly promotes kidney injury, because taxonomic changes alone cannot define microbial function. However, recent evidence from the longitudinal CKD-Microbiome Study showed that CKD-associated gut dysbiosis is accompanied by altered microbial functional capacity, increased uremic toxin biosynthesis, and CKD progression, supporting the possibility that injury-associated taxa may contribute to renal progression through toxin-related and inflammation-related pathways (Wehedy et al., 2021; Laiola et al., 2025).

In contrast, the taxa negatively associated with renal injury indices, including Akkermansia, Parasutterella, Allobaculum, and Parabacteroides, may reflect a relatively protective microbial signature. Akkermansia is closely associated with the intestinal mucus layer and epithelial barrier homeostasis, and previous CKD-specific evidence has shown that Akkermansia muciniphila may ameliorate renal interstitial fibrosis through the gut-renal axis by improving intestinal microecology and reducing intestinal mucosal barrier damage. Therefore, the negative association between Akkermansia and renal fibrosis-related indices in the present study is consistent with the concept that preservation of mucus-associated and barrier-supporting bacteria may be beneficial in the context of gut-kidney axis dysfunction. Parabacteroides has also received increasing attention as a potential next-generation probiotic genus involved in host metabolic regulation, immune modulation, and inflammatory homeostasis (Liu J. et al., 2025). Thus, the reduction or negative association of these potentially beneficial taxa may indicate a loss of protective microbial functions during CKD progression.

Mechanistically, gut microbiota dysbiosis may contribute to CKD progression through multiple metabolite- and inflammation-dependent pathways. Disruption of the intestinal barrier may facilitate the translocation of bacterial components and gut-derived toxic metabolites into the circulation, thereby promoting systemic inflammation, oxidative stress, tubular injury, and renal fibrosis. In addition to gut-derived uremic toxins such as indoxyl sulfate and p-cresyl sulfate, alterations in short-chain fatty acids, bile acid metabolism, and tryptophan-derived indole metabolites may also participate in the regulation of intestinal barrier integrity, immune activation, and renal pathological remodeling. Recent evidence further supports the importance of microbiota-derived short-chain fatty acids in maintaining intestinal homeostasis and restraining inflammatory responses, as shown by the study that Pingwei Powder alleviated high-fat diet-induced colonic inflammation by modulating microbial metabolites, particularly short-chain fatty acids (Liu T. et al., 2025). Inflammatory signaling may represent another key link between gut dysbiosis and kidney injury. A recent study demonstrated that Plantago asiatica L. extract alleviated hyperuricemia-associated renal injury by modulating gut microbiota and inhibiting NLRP3 inflammasome activation (Ou et al., 2026b). Similarly, Huopu Xialing decoction was reported to mitigate influenza A-induced pulmonary injury by inhibiting METTL3-Nlrp3(m6A)-mediated NLRP3 inflammasome activation, supporting the broader concept that gut microbiota can regulate distal organ inflammation through inflammasome-related pathways (Ou et al., 2026a). These studies, together with our findings, suggest that SIRT3 deficiency-associated gut dysbiosis may aggravate CKD progression not only through intestinal barrier impairment and microbial compositional changes, but also through microbiota-derived metabolites and inflammasome-related inflammatory signaling.

Taken together, the *in vivo* and *in vitro* findings indicate that SIRT3 exerts a protective role during CKD-related pathological progression (Lu et al., 2023). SIRT3 deficiency alone did not produce overt abnormalities under basal conditions; however, in the setting of adenine-induced CKD, it further aggravated renal dysfunction, inflammatory responses, tubulointerstitial fibrosis, and intestinal mucosal barrier injury, while also being accompanied by more pronounced gut microbial alterations. Similarly, *in vitro* inhibition of SIRT3 enhanced TGF-β1-induced profibrotic responses and apoptosis in HK-2 cells. These results suggest that SIRT3 is more likely to function as a protective regulator under injury and stress conditions, thereby restraining CKD-associated pathological progression. It should also be noted that the gut microbiota data in the present study mainly reflect CKD-associated microbial alterations in the context of SIRT3 deficiency; therefore, its independent role under basal conditions and its potential amplifying effect during disease progression require further investigation. In addition, because oxidative stress, mitochondrial dysfunction, and downstream molecular events were not directly examined, and the *in vitro* experiments relied primarily on pharmacological inhibition, further studies incorporating genetic approaches and causal validation are needed to more precisely define the role of SIRT3 in CKD progression.

## 5 Conclusions

In conclusion, the present study demonstrates that SIRT3 plays a protective role in chronic kidney disease. SIRT3 deficiency markedly aggravated adenine-induced renal dysfunction, inflammatory responses, tubulointerstitial fibrosis, intestinal mucosal barrier injury, and gut microbiota dysbiosis *in vivo*, while pharmacological inhibition of SIRT3 further enhanced TGF-β1-induced profibrotic responses and apoptosis in HK-2 cells *in vitro*. These findings indicate that SIRT3 deficiency exacerbates CKD progression not only at the level of local renal injury, but also in association with gut–kidney axis-related abnormalities. Collectively, our results suggest that SIRT3 may represent a potential therapeutic target for CKD and provide a basis for further mechanistic studies on its role in renal fibrosis and gut–kidney axis dysfunction.

## Data Availability

The 16S rRNA sequencing data generated in this study have been deposited in the NCBI Sequence Read Archive (SRA) under BioProject accession number PRJNA1480153.
