# Supplementary material for: SIRT3 deficiency aggravates renal injury and fibrosis in chronic kidney disease and is associated with intestinal barrier dysfunction and gut microbiota dysbiosis
Source: Front Microbiol. 2026 Jul 13;17:1851246. doi: 10.3389/fmicb.2026.1851246 (PMC13402368; doi:10.3389/fmicb.2026.1851246)

**Supplementary Figure S1. Original Western blot images used for quantification**

**Panel 1. Collagen I**


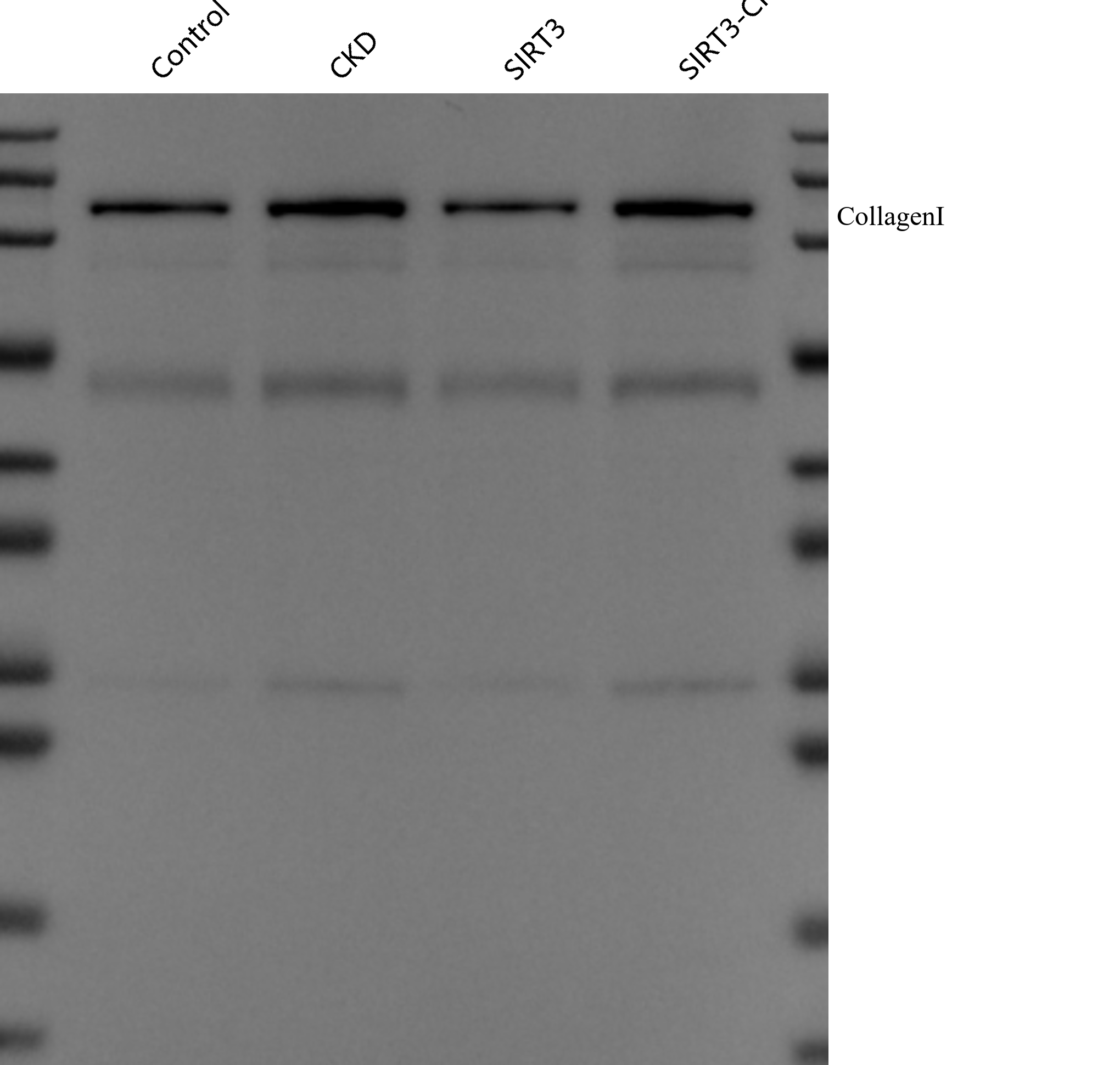


**Panel 2. Fibronectin**


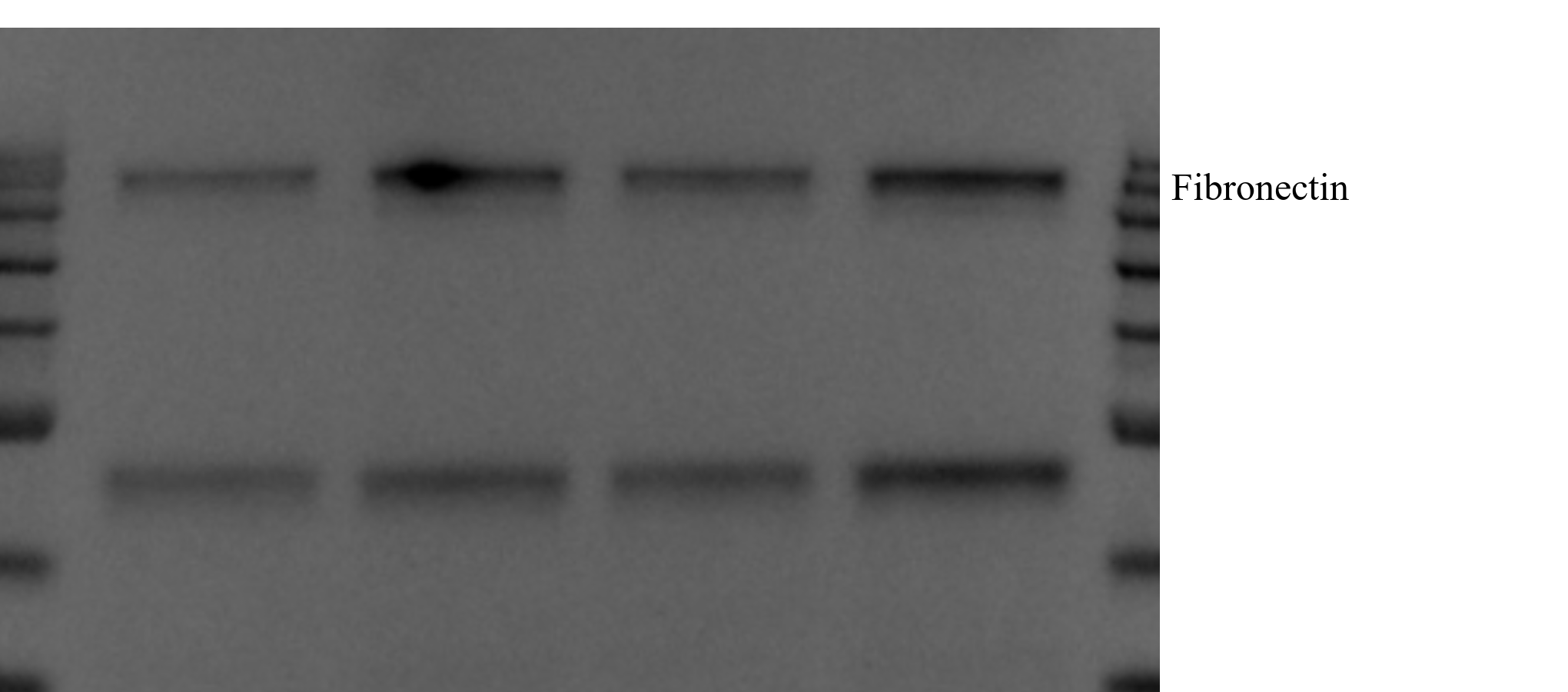


**Panel 3. Occludin**


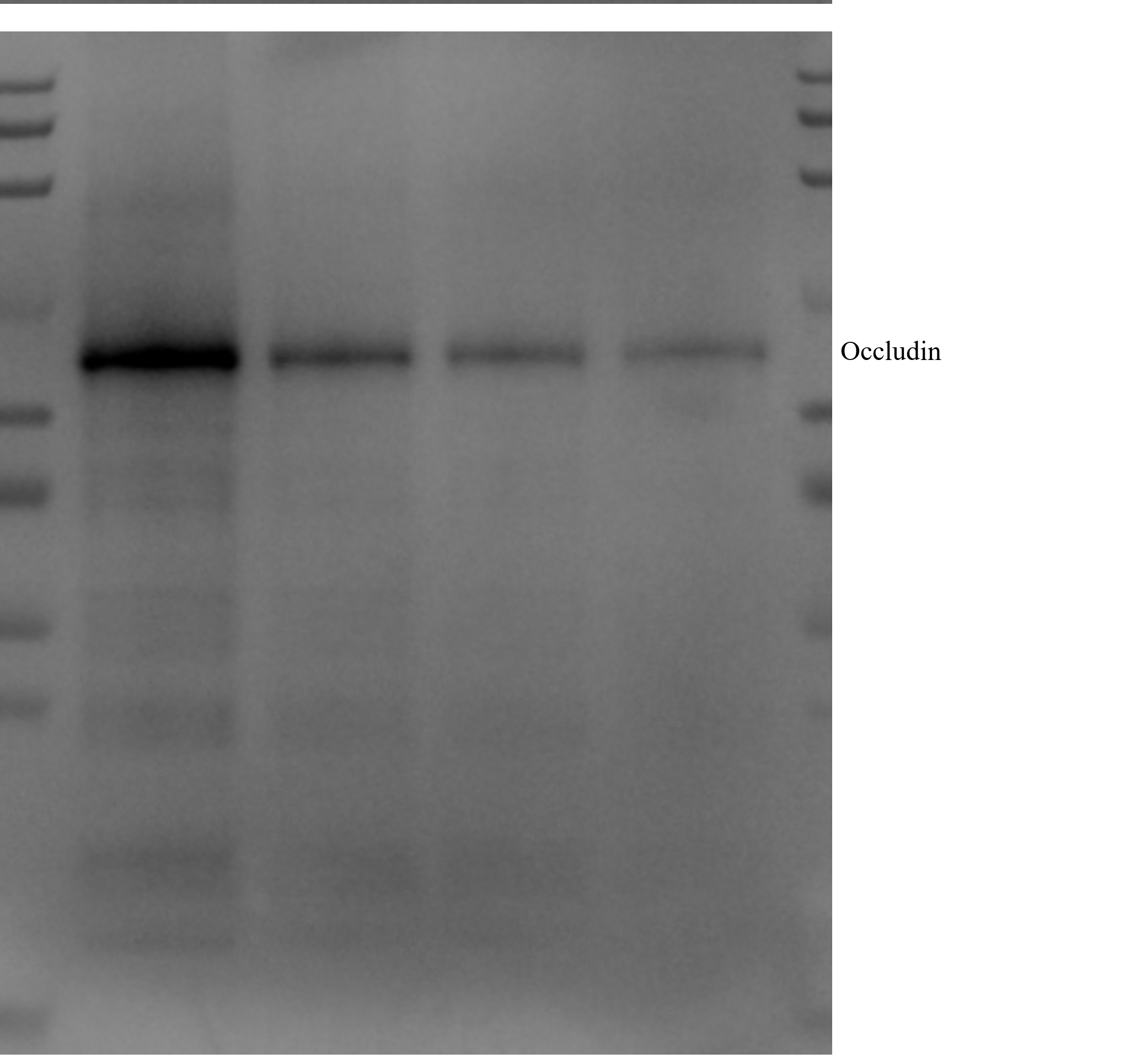


**Panel 4. SIRT3**


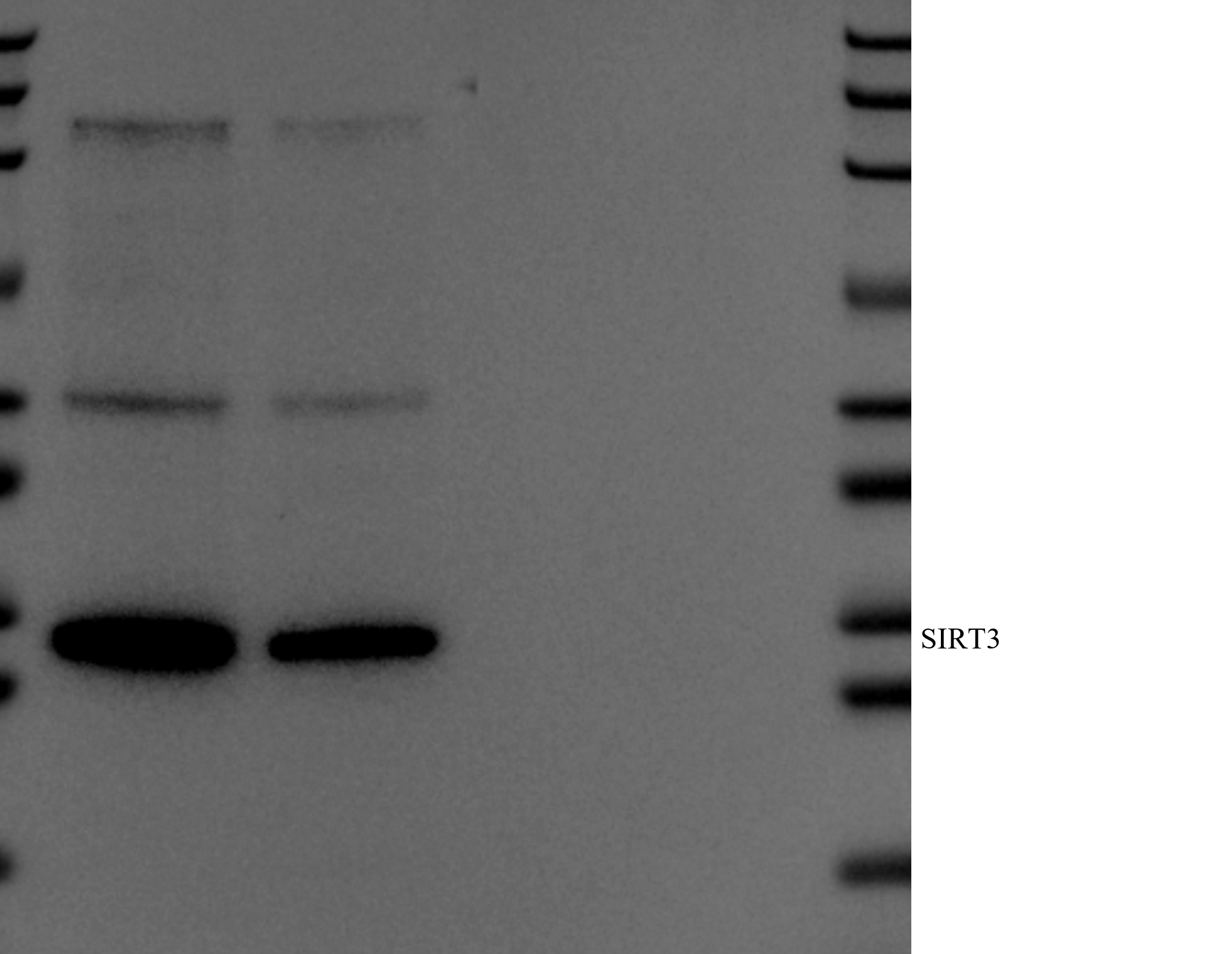


**Panel 5. ZO-1**


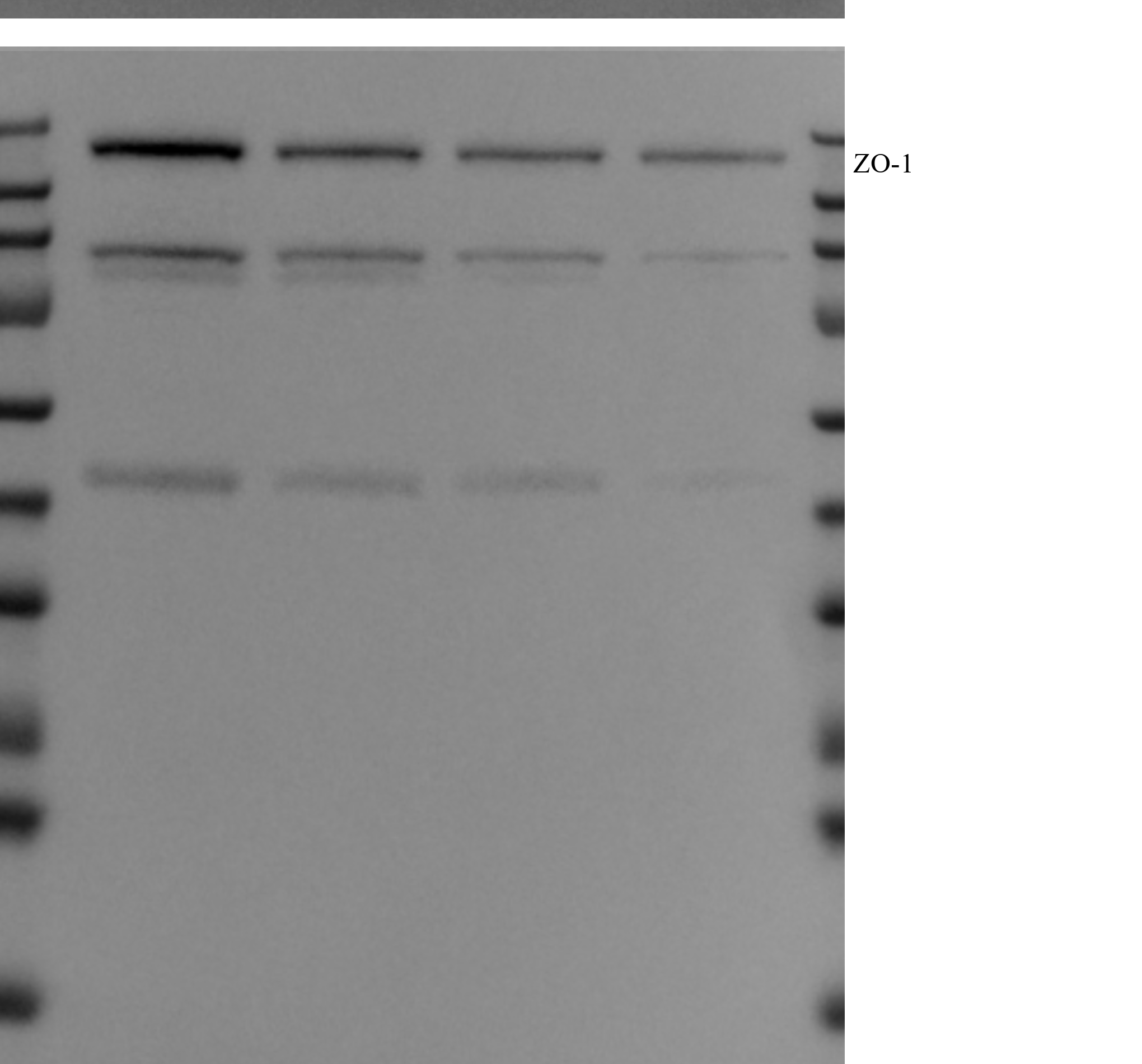


**Panel 6. α-SMA**


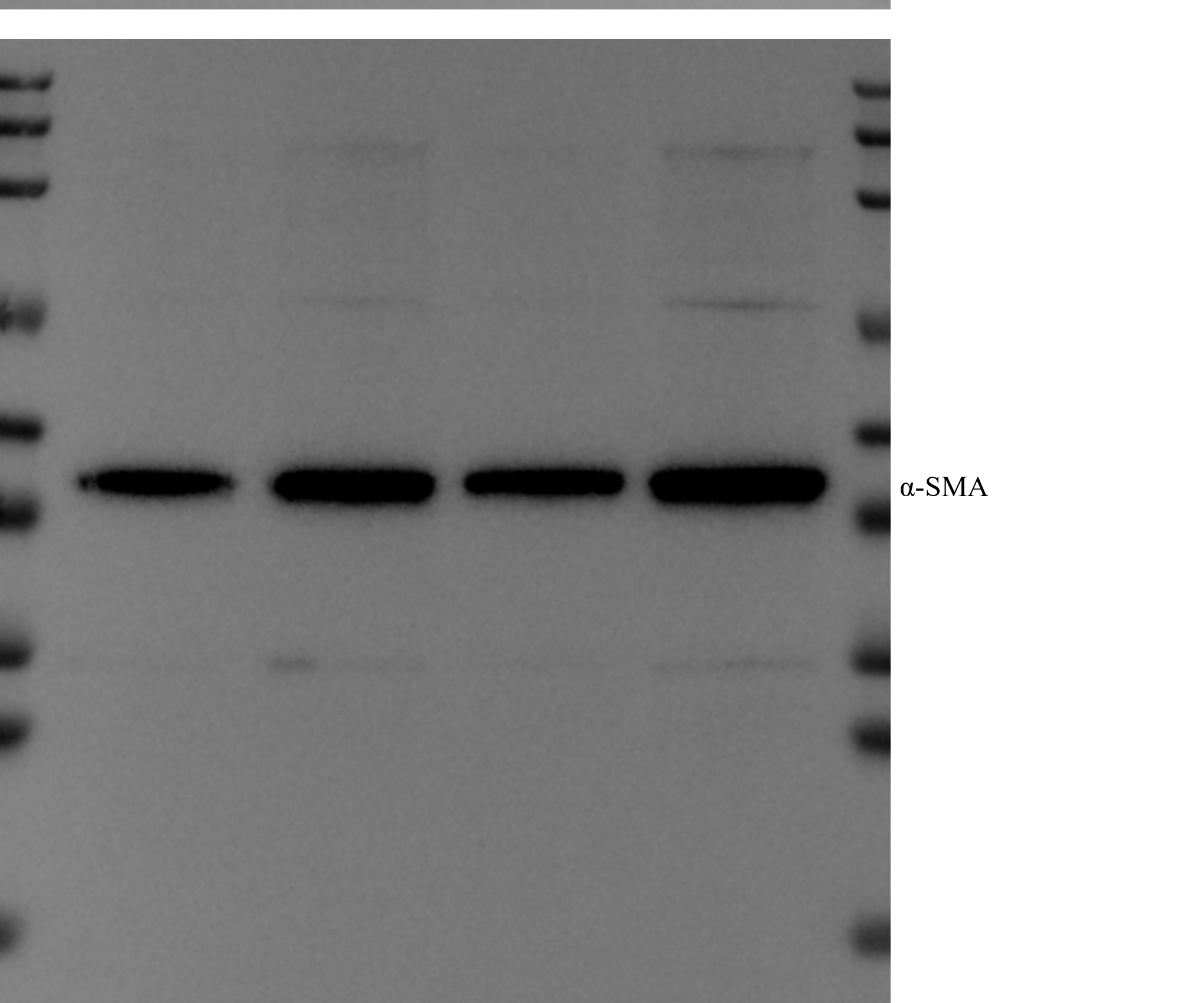


**Panel 7. β-Actin**


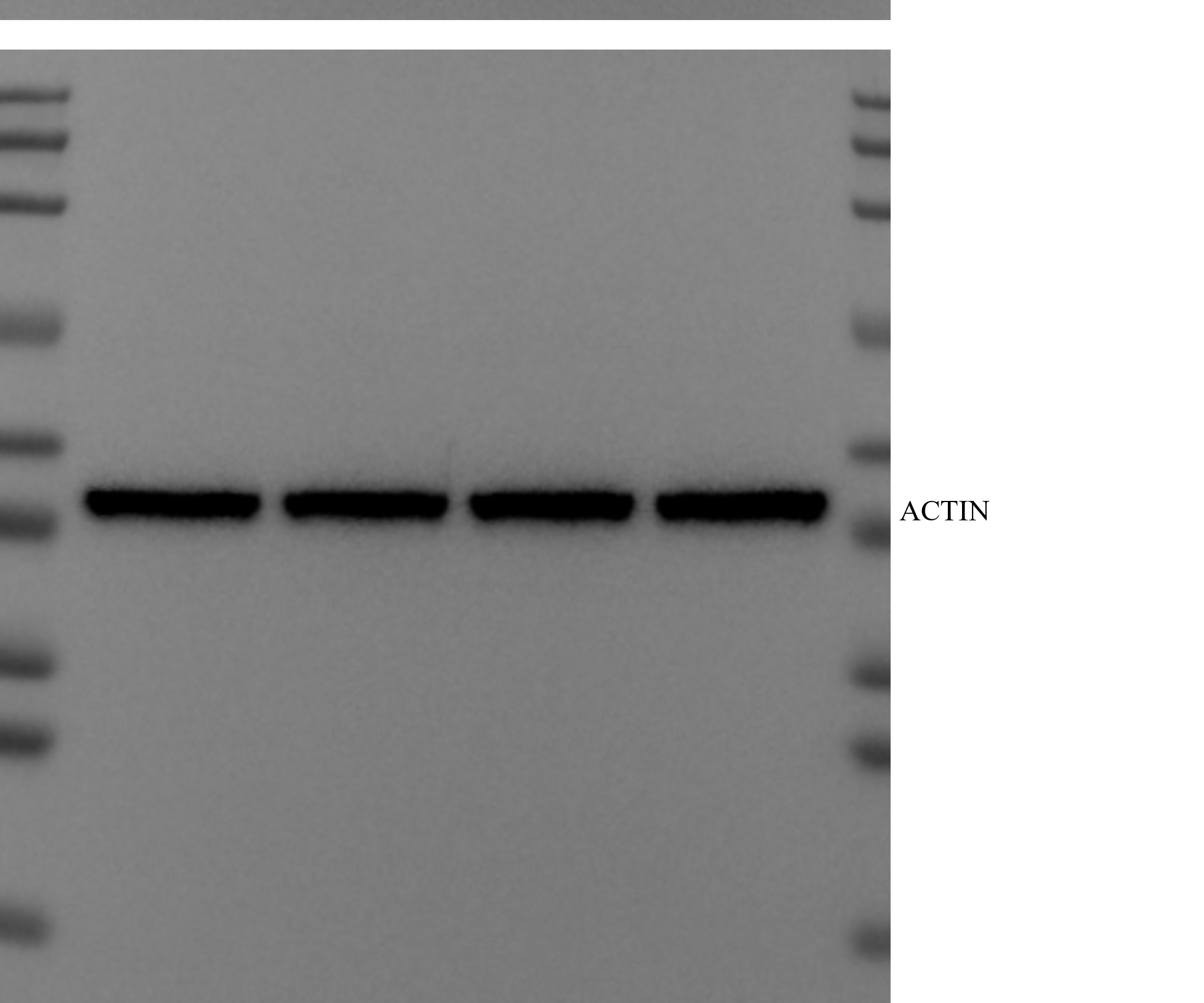

Supplement: Supplementary file 3 [file Data_Sheet_1.docx]
